# Supplementary material for: Plasma exosomes from individuals with type 2 diabetes drive breast cancer aggression in patient-derived organoids
Source: Commun Biol. 2025 Aug 26;8:1276. doi: 10.1038/s42003-025-08663-y (PMC12381303; doi:10.1038/s42003-025-08663-y)
Supplement: Supplementary file 1 — Supplementary Information [file 42003_2025_8663_MOESM1_ESM.pdf]

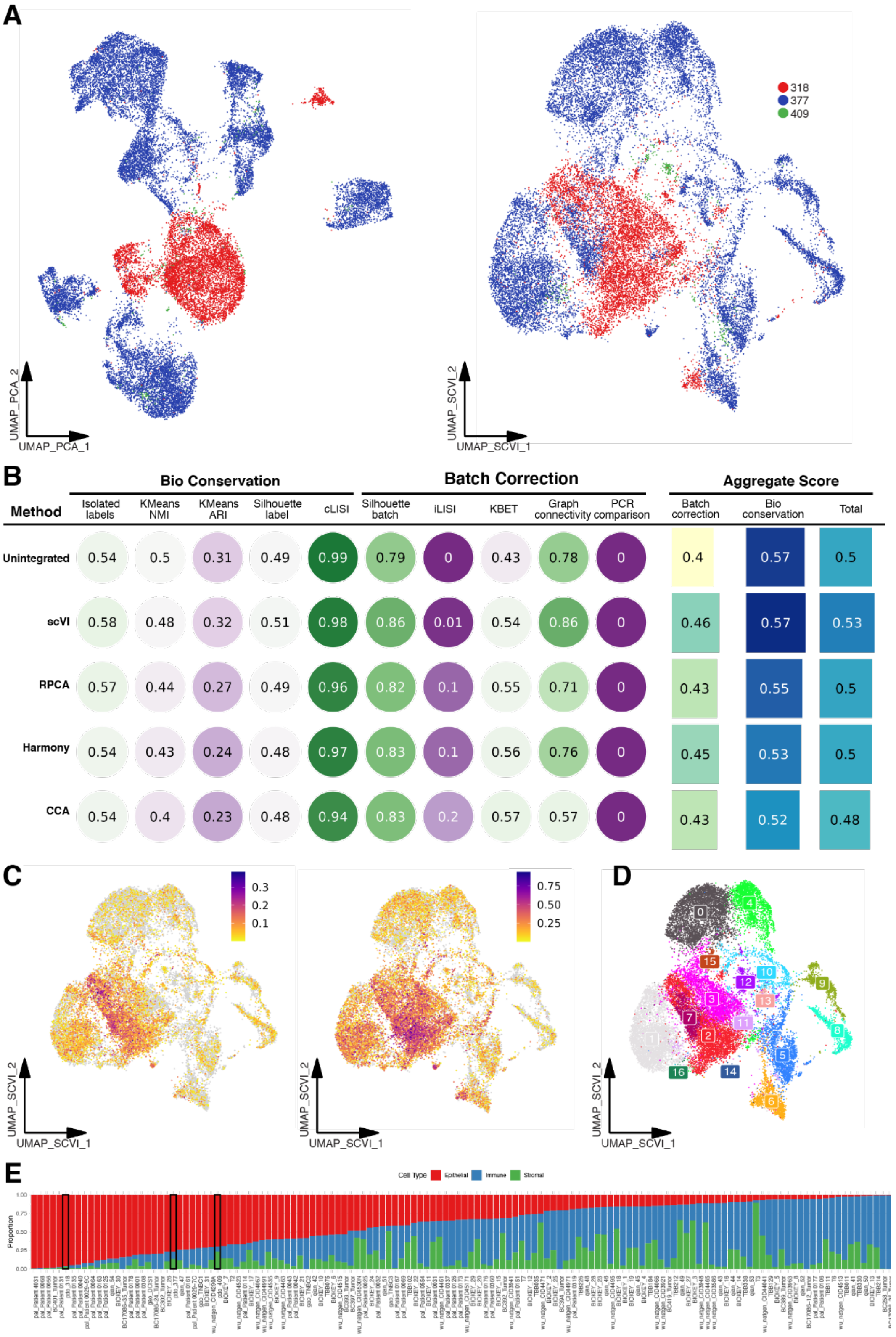

Fig S1. scRNAseq metrics and non-integrated data.

A. UMAP visualizations of PDOs colored by patient, showing integrated (left) and scVI-integrated (right) data. **B.** Benchmarking results assessing batch correction performance across dataset. **C.** UMAP

visualizations colored by module score of COSMIC CGC (left) and GOBP_MAMMARY_EPITHELIAL_PROLIFATION (right) gene expression. **D**. UMAP visualization of scVI-integrated data colored by Louvain clusters. **E.** Barplot showing proportion of cells identified as epithelial, stromal, or immune in benchmarking atlas. Distributions of PDOs boxed.

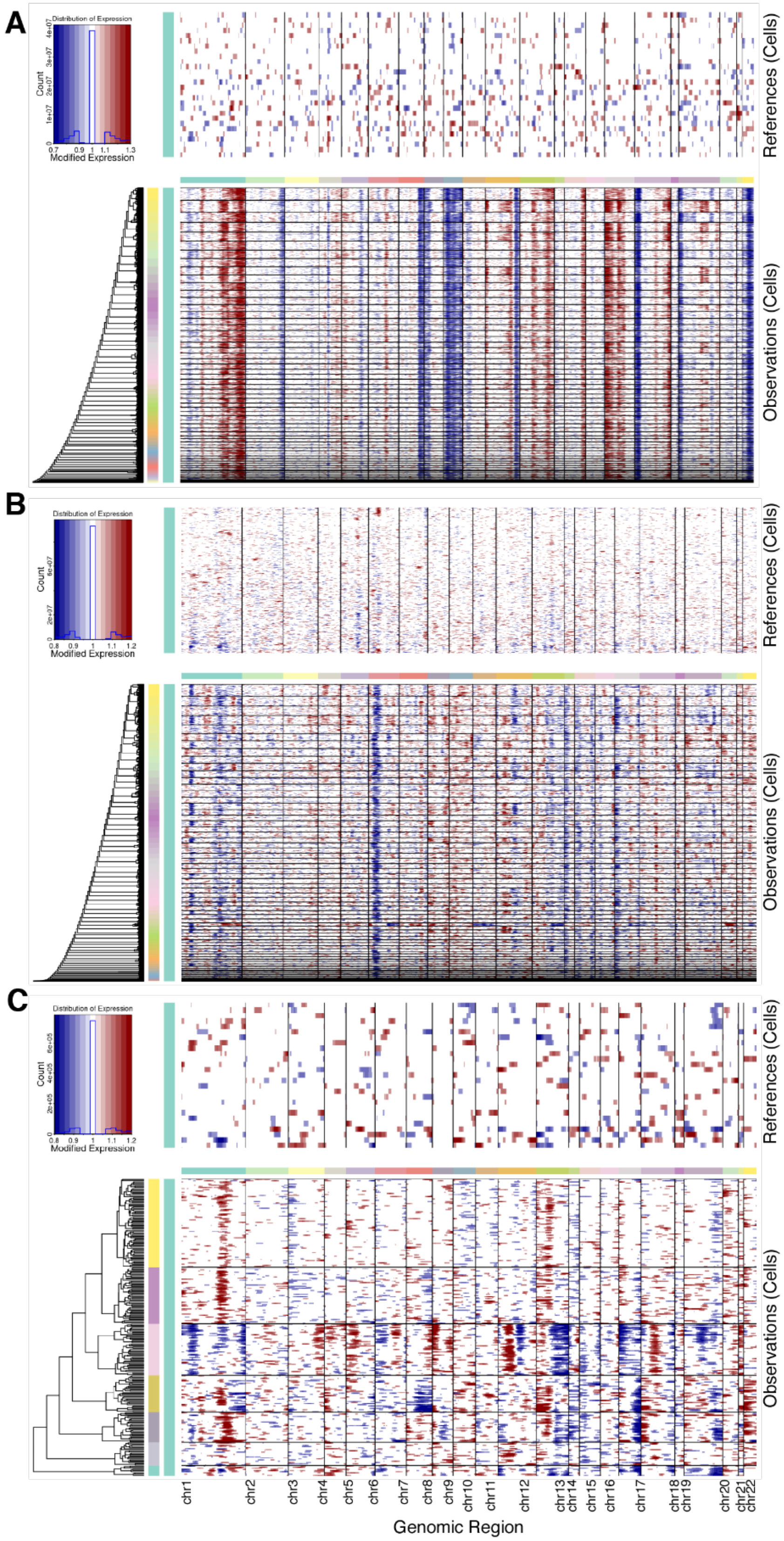

Fig S2. Identification of malignant cells.

A/B/C. InferCNV heatmaps of all malignant cells for patients 318 (**A**), 377 (**B**) and 409 (**C**). Immune cells used as reference on an individual patient basis.

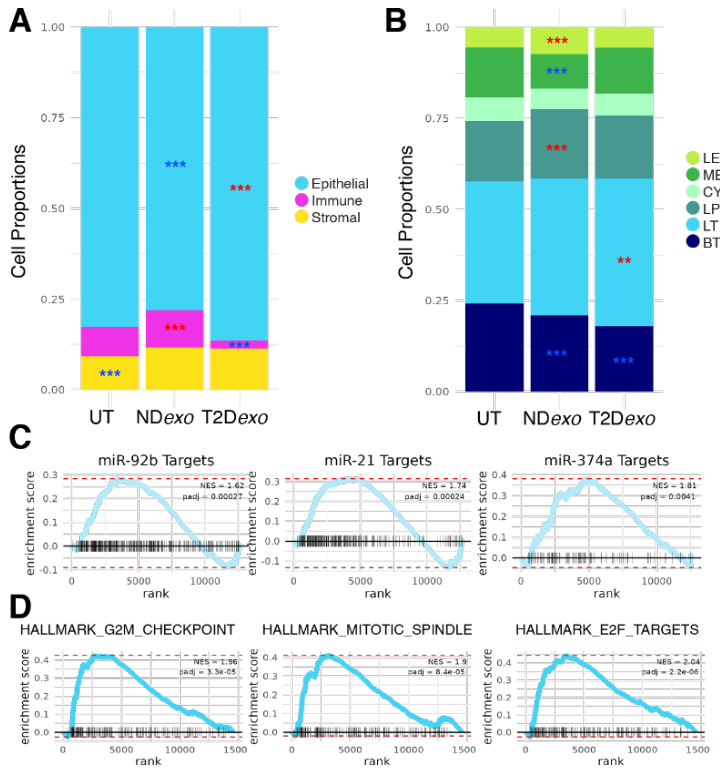

Fig S3. Relative abundance and cell type dynamics.

A. Relative proportion of coarse annotations per treatment. Note uniformity of stromal compartment across treatments. Significance calculated via binomial linear regression model, ***<0.001, **<0.01, *<0.05. **B.** Relative proportion of all epithelial and tumor subclones. Significance calculated via binomial linear regression model, ***<0.001, **<0.01, *<0.05. **C/D.** GSEA enrichment plots for representative gene sets upregulated in LT1 (**C**) or LT3 (**D**).

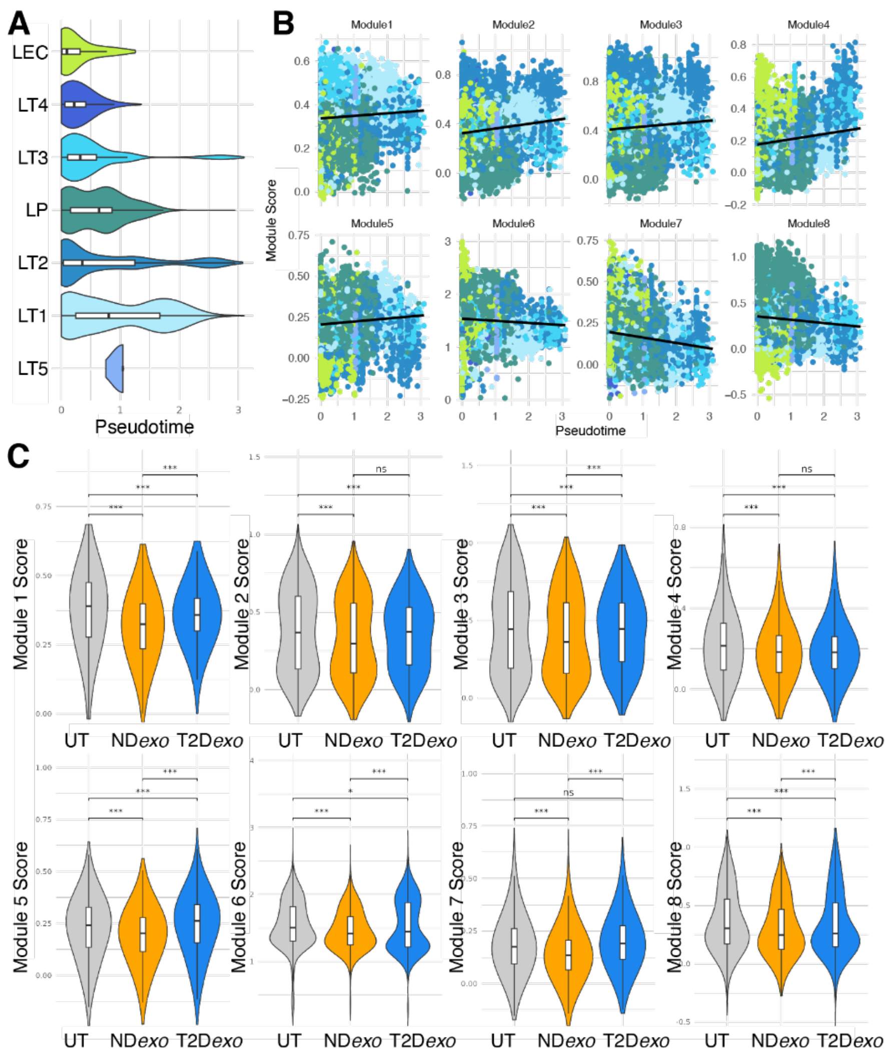

Fig S4. Pseudotime analysis and module characterization.

A. Violin plots of pseudotime calculated for luminal-like epithelial cells per cell type. **B.** Scatter plots of module gene set expression as a function of pseudotime. Dot color denotes epithelial cell subtype assigned to cells. Black line showing linear regression of best fit. **C.** Violin plots of module expression in luminal-like epithelial cells per treatment. Significance calculated via linear mixed effects model, ***<0.001, **<0.01, *<0.05.

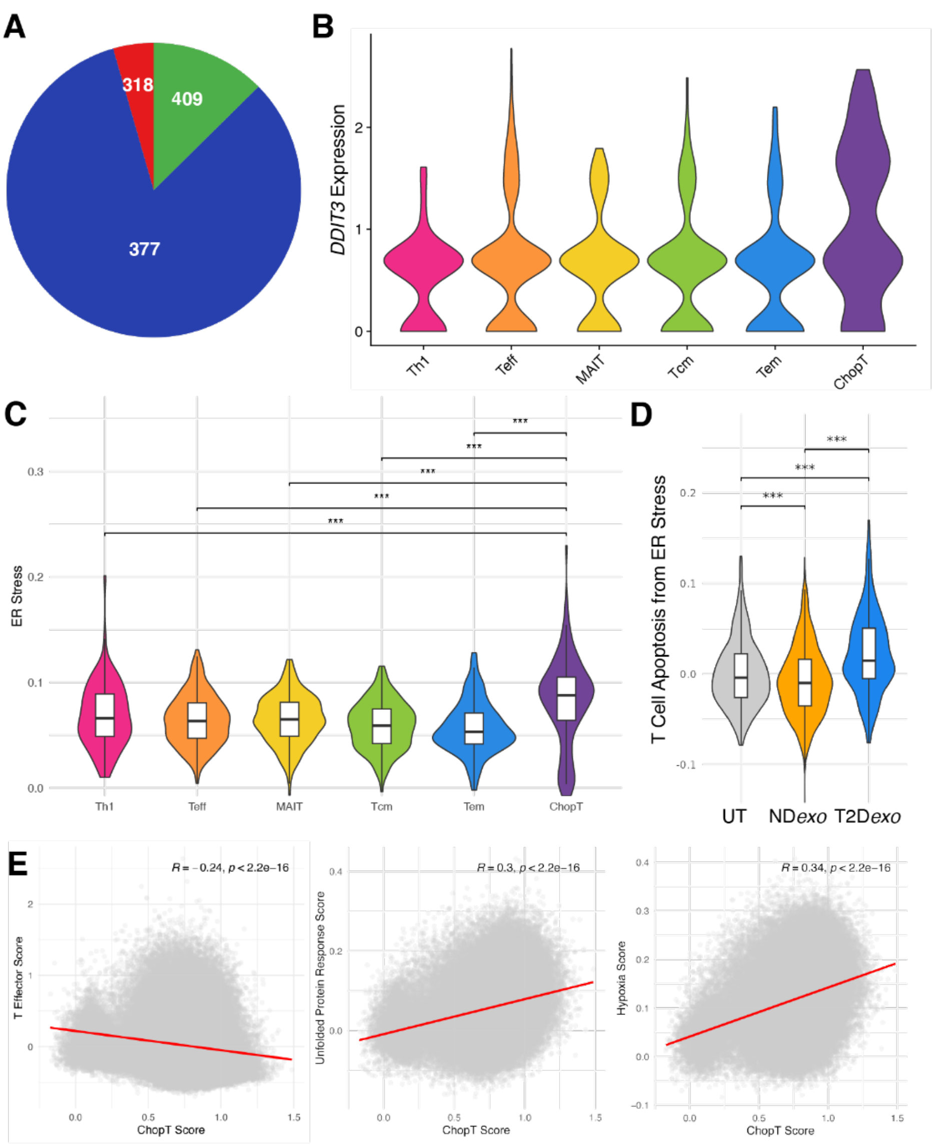

Fig S5. Characterization of ChopT cells.

A. Pie chart of the distribution of ChopT cells across patients, demonstrating its conservation. **B.** Violin plots of *DDIT3* expression across T cell states. **C.** Violin plots of expression of GOBP_RESPONSE_TO_ENDOPLASMIC_RETICULUM_STRESS across T cell states. Significance calculated via linear mixed effects model, ***<0.001, **<0.01, *<0.05. **D.** Violin plots of expression of GOBP_INTRINSIC_APOPTOTIC_SIGNALING_PATHWAY_IN_RESPONSE_TO_ENDOPLASMIC_RETICULUM_STRESS in T cells across treatment groups. Significance calculated via linear mixed effects model, ***<0.001, **<0.01, *<0.05. **E.** Scatter plots of ChopT module score versus effector score (left), UPR score (middle), and hypoxia (right) across the pan-breast cancer immune cell atlas. Red lines indicate lines of best fit. Spearman correlation coefficients and p-values shown.

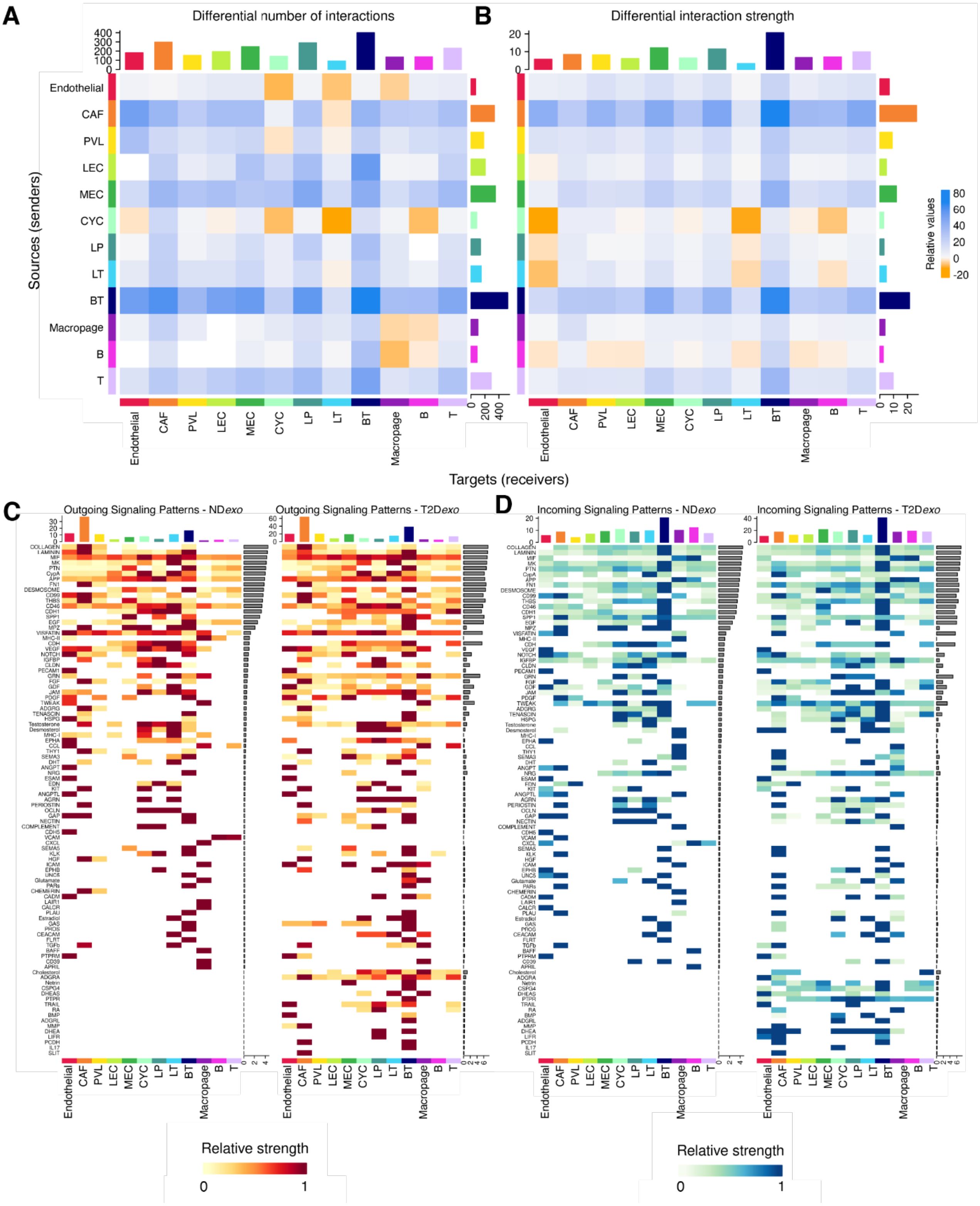

Fig S6. Cell-cell communication networks.

A. Heatmap showing the number of interactions among different cell types. The blue (orange) colored edges represent increased (decreased) signaling in T2D*exo* compared to ND*exo*-PDOs. **B**. Heatmap showing the differential interaction strength among different cell types. The blue (orange) colored edges

represent increased (decreased) interaction strength in T2D*exo* compared to ND*exo*-PDOs. **C.** Comparison heatmap of patterns of ingoing communication in ND*exo*-PDOs (left) and T2D*exo*-PDOs (right). **D.** Comparison heatmap of patterns of outgoing communication in ND*exo*-PDOs (left) and T2D*exo*-PDOs (right).

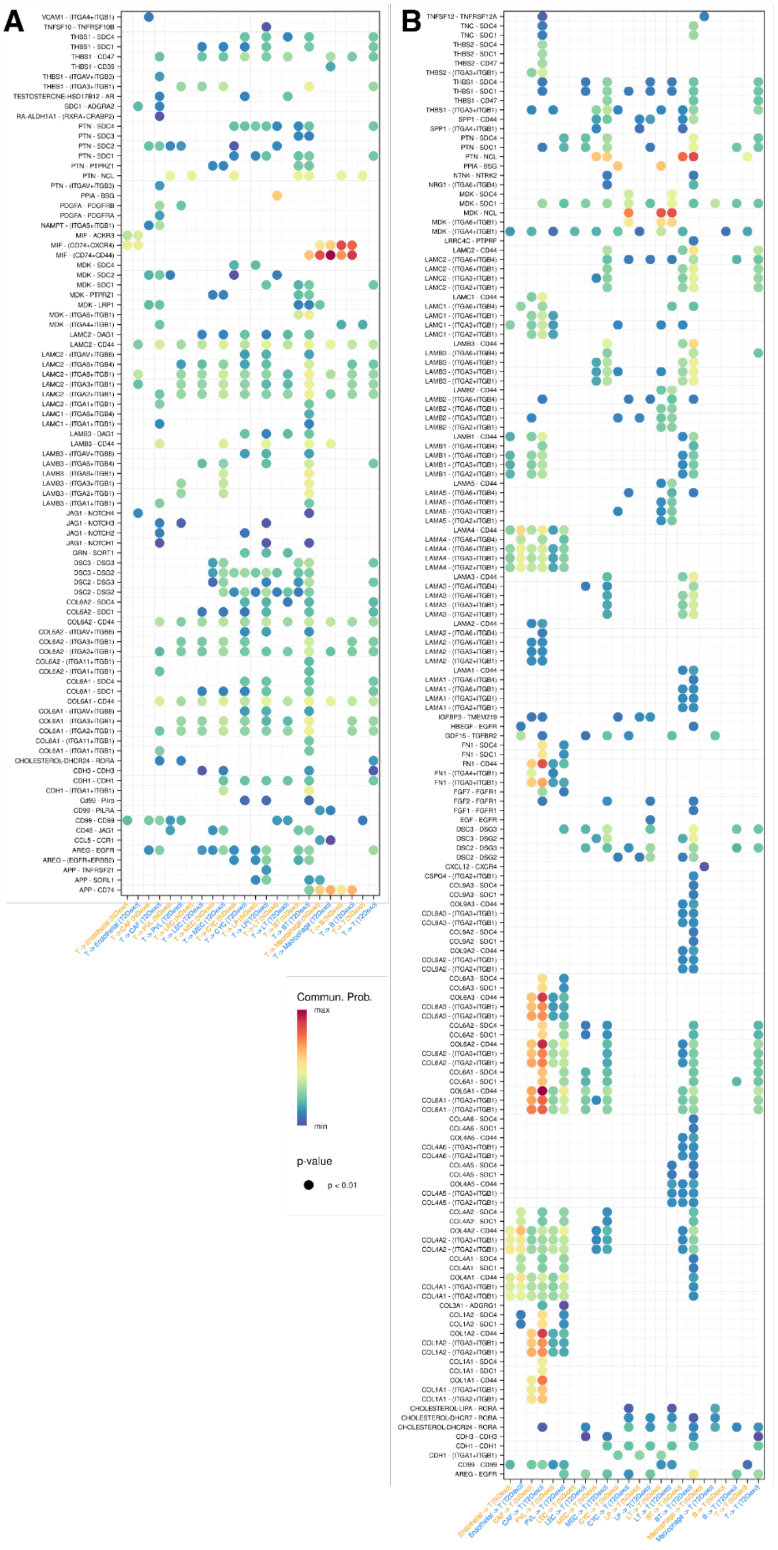

Fig S7. T cell-specific communication networks.

A/B. Dot plot of expression levels of significant ($p < 0.01$) ligand-receptor pairs from all senders to (**A**) and all receivers from (**B**) T cells. *P* values are computed from a one-sided permutation test according to CellChat.

Supplementary Data 1. Clinicopathological characteristics of patient tumors.

This table summarizes the age at diagnosis, tumor size, histological type, tumor grade, lymph node involvement, TNM stage, hormone receptor status (ER, PR), HER2 status, and Ki-67 proliferation index for each patient included in the study.

Supplementary Data 2. Marker genes for broad cell types identified in PDOs.

This table lists the marker genes used to identify and characterize broad cell types maintained in PDOs. The table includes gene names per cell type and associated statistics.

Supplementary Data 3. GSEA results comparing T2D*exo* vs. ND*exo* across all cells.

This table lists the significant gene sets (adjusted p-value < 0.05) identified by GSEA when comparing all cells captured within T2D*exo*-PDOs vs. ND*exo*-PDOs. The table includes the gene set names and associated statistics.

Supplementary Data 4. Differentially expressed genes in T2D*exo* vs. ND*exo* across all cells.

This table lists the differentially expressed genes identified in T2D*exo*-PDOs compared to ND*exo*-PDOs across all cell types with associated statistics.

Supplementary Data 5. Circularity measurements of PDOs.

This table summarizes the area, perimeter, and circularity measurements of individual PDOs. Singlets were removed during thresholding.

Supplementary Data 6. Composite gene signature for survival analysis.

This table lists top genes upregulated in either T2D*exo*-treated PDOs or in ND*exo*-treated PDOs, which together comprise the composite signature applied to TCGA and METABRIC cohorts.

Supplementary Data 7. Marker genes for epithelial clusters identified in PDOs.

This table lists the marker genes used to identify and characterize epithelial and tumor subclones maintained in PDOs. The table includes gene names per cell type and associated statistics.

Supplementary Data 8. GSEA results comparing LT1 vs. all other luminal epithelial cells.

This table lists the significant gene sets (adjusted p-value < 0.05) identified by GSEA when comparing LT1 cells compared to all other luminal-like epithelial cells. The table includes the gene set names and associated statistics.

Supplementary Data 9. Differentially expressed genes along pseudotime.

This table presents the results of graph autocorrelation analysis, identifying genes with significant expression changes along the pseudotime trajectory. The table includes gene names and associated statistics.

Supplementary Data 10. Genes comprising co-regulated modules in pseudotime.

This table lists the genes grouped into distinct modules of co-regulation. Each module represents a cluster of genes with synchronized expression patterns along the pseudotime trajectory, reflecting their potential involvement in specific biological processes during tumor progression.

Supplementary Data 11. Marker genes for immune clusters identified in PDOs.

This table lists the marker genes used to identify and characterize immune and T cell states maintained in PDOs. The table includes gene names per cell type and associated statistics.

Supplementary Data 12. Differentially expressed genes in T2D*exo* vs. ND*exo* across immune cells.

This table lists the differentially expressed genes identified in T2D*exo*-PDOs compared to ND*exo*-PDOs across immune cell types with associated statistics.

Supplementary Data 13. GSEA results comparing T2D*exo* vs. ND*exo* across immune cells.

This table lists the significant gene sets (adjusted p-value < 0.05) identified by GSEA when comparing immune cells captured within T2D*exo*-PDOs vs. ND*exo*-PDOs. The table includes the gene set names and associated statistics.

Supplementary Data 14. Enrichment results on branch point via K2Taxonomer.

This table summarizes the significantly upregulated gene sets (p-value < 0.05) identified during K2Taxonomer analysis of ChopT vs. normal development branch point. The table includes the gene set names and associated statistics.

Supplementary Data 15. Intercellular communication inferred from ligand-receptor interactions.

This table provides an overview of the cell-cell communication networks within T2D*exo*-PDOs and ND*exo*-PDOs as inferred from ligand-receptor interactions. This table lists identified ligand-receptor pairs, their pathway annotations, the cell types involved in these interactions, and associated statistics.
