# Supplementary material for: Plasma exosomes from individuals with type 2 diabetes drive breast cancer aggression in patient-derived organoids
Source: Commun Biol. 2025 Aug 26;8:1276. doi: 10.1038/s42003-025-08663-y (PMC12381303; doi:10.1038/s42003-025-08663-y)
Supplement: Supplementary file 2 — Description of Additional Supplementary Files [file 42003_2025_8663_MOESM2_ESM.pdf]

Description of Additional Supplementary Files

File name: Supplementary Data 1.

Description: Clinicopathological characteristics of patient tumors. This table summarizes the age at diagnosis, tumor size, histological type, tumor grade, lymph node involvement, TNM stage, hormone receptor status (ER, PR), HER2 status, and Ki-67 proliferation index for each patient included in the study.

File name: Supplementary Data 2.

Description: Marker genes for broad cell types identified in PDOs. This table lists the marker genes used to identify and characterize broad cell types maintained in PDOs. The table includes gene names per cell type and associated statistics.

File name: Supplementary Data 3.

Description: GSEA results comparing T2Dexo vs. NDexo across all cells. This table lists the significant gene sets (adjusted p-value < 0.05) identified by GSEA when comparing all cells captured within T2Dexo-PDOs vs. NDexo-PDOs. The table includes the gene set names and associated statistics.

File name: Supplementary Data 4.

Description: Differentially expressed genes in T2Dexo vs. NDexo across all cells. This table lists the differentially expressed genes identified in T2Dexo-PDOs compared to NDexo-PDOs across all cell types with associated statistics.

File name: Supplementary Data 5.

Description: Circularity measurements of PDOs. This table summarizes the area, perimeter, and circularity measurements of individual PDOs. Singlets were removed during thresholding.

File name: Supplementary Data 6.

Description: Composite gene signature for survival analysis.This table lists top genes upregulated in either T2Dexo-treated PDOs or in NDexo-treated PDOs, which together comprise the composite signature applied to TCGA and METABRIC cohorts.

File name: Supplementary Data 7.

Description: Marker genes for epithelial clusters identified in PDOs. This table lists the marker genes used to identify and characterize epithelial and tumor subclones maintained in PDOs. The table includes gene names per cell type and associated statistics.

File name: Supplementary Data 8.

Description: GSEA results comparing LT1 vs. all other luminal epithelial cells. This table lists the significant gene sets (adjusted p-value < 0.05) identified by GSEA when comparing LT1 cells compared to all other luminal-like epithelial cells. The table includes the gene set names and associated statistics.

File name: Supplementary Data 9.

Description: Differentially expressed genes along pseudotime. This table presents the results of graph autocorrelation analysis, identifying genes with significant expression changes along the pseudotime trajectory. The table includes gene names and associated statistics.

File name: Supplementary Data 10.

Description: Genes comprising co-regulated modules in pseudotime. This table lists the genes grouped into distinct modules of co-regulation. Each module represents a cluster of genes with synchronized expression patterns along the pseudotime trajectory, reflecting their potential involvement in specific biological processes during tumor progression.

File name: Supplementary Data 11.

Description: Marker genes for immune clusters identified in PDOs. This table lists the marker genes used to identify and characterize immune and T cell states maintained in PDOs. The table includes gene names per cell type and associated statistics.

File name: Supplementary Data 12.

Description: Differentially expressed genes in T2Dexo vs. NDexo across immune cells. This table lists the differentially expressed genes identified in T2Dexo-PDOs compared to NDexo-PDOs across immune cell types with associated statistics.

File name: Supplementary Data 13.

Description: GSEA results comparing T2Dexo vs. NDexo across immune cells. This table lists the significant gene sets (adjusted p-value < 0.05) identified by GSEA when comparing immune cells captured within T2Dexo-PDOs vs. NDexo-PDOs. The table includes the gene set names and associated statistics.

File name: Supplementary Data 14.

Description: Enrichment results on branch point via K2Taxonomer. This table summarizes the significantly upregulated gene sets (p-value < 0.05) identified during K2Taxonomer analysis of ChopT vs. normal development branch point. The table includes the gene set names and associated statistics.

File name: Supplementary Data 15.

Description: Intercellular communication inferred from ligand-receptor interactions. This table provides an overview of the cell-cell communication networks within T2Dexo-PDOs and NDexo-PDOs as inferred from ligand-receptor interactions. This table lists identified ligand-receptor pairs, their pathway annotations, the cell types involved in these interactions, and associated statistics.
