# Supplementary material for: Plasma exosomes from individuals with type 2 diabetes drive breast cancer aggression in patient-derived organoids
Source: Commun Biol. 2025 Aug 26;8:1276. doi: 10.1038/s42003-025-08663-y (PMC12381303; doi:10.1038/s42003-025-08663-y)
Supplement: Supplementary file 4 — Reporting summary [file 42003_2025_8663_MOESM4_ESM.pdf]

nature portfolio

Corresponding author(s): Gerald V Denis

Last updated by author(s): May 1, 2025

Reporting Summary

Nature Portfolio wishes to improve the reproducibility of the work that we publish. This form provides structure for consistency and transparency in reporting. For further information on Nature Portfolio policies, see our Editorial Policies and the Editorial Policy Checklist.

Statistics

For all statistical analyses, confirm that the following items are present in the figure legend, table legend, main text, or Methods section.

n/a	Confirmed	
☐	☒	The exact sample size (*n*) for each experimental group/condition, given as a discrete number and unit of measurement
☐	☒	A statement on whether measurements were taken from distinct samples or whether the same sample was measured repeatedly
☐	☒	The statistical test(s) used AND whether they are one- or two-sided<br>*Only common tests should be described solely by name; describe more complex techniques in the Methods section.*
☐	☒	A description of all covariates tested
☐	☒	A description of any assumptions or corrections, such as tests of normality and adjustment for multiple comparisons
☐	☒	A full description of the statistical parameters including central tendency (e.g. means) or other basic estimates (e.g. regression coefficient) AND variation (e.g. standard deviation) or associated estimates of uncertainty (e.g. confidence intervals)
☐	☒	For null hypothesis testing, the test statistic (e.g. *F*, *t*, *r*) with confidence intervals, effect sizes, degrees of freedom and *P* value noted<br>*Give P values as exact values whenever suitable.*
☒	☐	For Bayesian analysis, information on the choice of priors and Markov chain Monte Carlo settings
☒	☐	For hierarchical and complex designs, identification of the appropriate level for tests and full reporting of outcomes
☐	☒	Estimates of effect sizes (e.g. Cohen's *d*, Pearson's *r*), indicating how they were calculated

Our web collection on statistics for biologists contains articles on many of the points above.

Software and code

Policy information about availability of computer code

Data collection	Quality and quantity of the amplified cDNA were assessed using an Angilent Bioanalyzer 2100 High Sensitivity Kit. Libraries were then multiplexed and sequenced on an Illumina NextSeq 2000 platform (RRID:SCR_023614), aiming for a depth of approximately 30,000 reads per cell, which provides sufficient coverage to detect most expressed genes.
Data analysis	Transcriptomic data were mapped to the human reference genome (GRCh38.p14; RRID:SCR_006553) and assigned to individual cells of origin according to cell-specific barcodes using the Cell Ranger pipeline version 6.0.1 (10X Genomics; RRID:SCR_017344). Filtering, feature selection, clustering, and other secondary analyses were performed using Seurat v5.0.1 (RRID:SCR_016341)64. Initial preprocessing included the removal of doublets using the DoubletFinder v2.0.4 pipeline (RRID:SCR_018771). The data were further filtered to exclude cells with high mitochondrial gene expression (>20%) and aberrant unique feature counts (<100, >9000), followed by normalization and identification of highly variable features using scTransform v0.4.1 (RRID:SCR_022146)65. Dimensionality reduction was carried out via Principal Component Analysis using 50 dimensions, followed by clustering using the graph-based Louvain method with a resolution of 0.5 to identify distinct cellular populations. Cell types within these clusters were then automatically annotated using the SingleR pipeline (RRID:SCR_023120)17, referencing an established comprehensive single cell atlas of breast cancer patient samples18. To harmonize the data across patients and mitigate batch effects, we performed dataset integration using scVI14 . This methodology was selected over alternatives based on k-nearest-neighbor batch effect test, integration local inverse Simpson's index, average silhouette width, and principal component regression comparison from single cell integration benchmarking (scIB) analysis.

For manuscripts utilizing custom algorithms or software that are central to the research but not yet described in published literature, software must be made available to editors and reviewers. We strongly encourage code deposition in a community repository (e.g. GitHub). See the Nature Portfolio guidelines for submitting code & software for further information.

Data

Policy information about availability of data

All manuscripts must include a data availability statement. This statement should provide the following information, where applicable:

- Accession codes, unique identifiers, or web links for publicly available datasets
- A description of any restrictions on data availability
- For clinical datasets or third party data, please ensure that the statement adheres to our policy

Data are publicly available at GSE302054.

Research involving human participants, their data, or biological material

Policy information about studies with human participants or human data. See also policy information about sex, gender (identity/presentation), and sexual orientation and race, ethnicity and racism.

Reporting on sex and gender	Sex was self reported
Reporting on race, ethnicity, or other socially relevant groupings	Adult male and female participants were recruited, ages 21 - 80 years. Race and ethnicity and other socially relevant characteristics were not collected.
Population characteristics	Men and Women, 21 - 80 years old, with a Type 2 diabetes diagnosis
Recruitment	Clinical coordinating staff or medical providers approached potential subjects in the BUMC Diabetes Outpatient Clinic. Subjects visit the diabetes outpatient clinic for regular appointments, which provided a convenient time for the proposed blood draw (5 cc). Upon completion of the 5cc blood draw from the antecubital vein, the sample of EDTA-anticoagulated blood was retrieved in a timely way by the clinical coordinator, for transport in biohazard-protection containment to the biochemistry laboratory in basic science space where the plasma will be prepared. The involvement of the subject in the study ended once the blood draw was completed.
Ethics oversight	Boston University (BU) Institutional Review Board (IRB) approved this study and carried out oversight; Protocol Number: H-42179

Note that full information on the approval of the study protocol must also be provided in the manuscript.

Field-specific reporting

Please select the one below that is the best fit for your research. If you are not sure, read the appropriate sections before making your selection.

☒ Life sciences ☐ Behavioural & social sciences ☐ Ecological, evolutionary & environmental sciences

For a reference copy of the document with all sections, see nature.com/documents/nr-reporting-summary-flat.pdf

Life sciences study design

All studies must disclose on these points even when the disclosure is negative.

Sample size	Patient derived organoids were generated from estrogen receptor-positive breast tumor resection samples from three nondiabetic patients
Data exclusions	These donors were chosen based on a preliminary screen of 15 patients where the subjects selected had the least medical/drug management
Replication	While patient-derived organoid (PDO) 318 showed lower immune cell abundance compared to PDO377 and PDO409, the variation observed across the three PDOs likely reflects real interpatient differences in tumor-immune composition—a feature well-documented in breast cancer. To contextualize these findings, we benchmarked our data against our recently assembled breast cancer single cell atlas, confirming that the cellular proportions in our PDOs fall within the spectrum observed in primary breast tumors. To control for variability across PDO lines, we explicitly model patient batch as a latent variable in our statistical framework. This approach, in which tumor, stromal, and immune populations are preserved in PDOs, represents a substantial advancement over traditional methods, which fail to maintain native breast TIL populations, and provides a comprehensive platform for studying immune-tumor interactions in breast cancer.
Randomization	n/a
Blinding	n/a

Reporting for specific materials, systems and methods

We require information from authors about some types of materials, experimental systems and methods used in many studies. Here, indicate whether each material, system or method listed is relevant to your study. If you are not sure if a list item applies to your research, read the appropriate section before selecting a response.

Materials & experimental systems

n/a	Involved in the study
☒	☐ Antibodies
☒	☐ Eukaryotic cell lines
☒	☐ Palaeontology and archaeology
☒	☐ Animals and other organisms
☒	☐ Clinical data
☒	☐ Dual use research of concern
☒	☐ Plants

Methods

n/a	Involved in the study
☐	☐ ChIP-seq
☐	☒ Flow cytometry
☐	☐ MRI-based neuroimaging

Plants

Seed stocks	*Report on the source of all seed stocks or other plant material used. If applicable, state the seed stock centre and catalogue number. If plant specimens were collected from the field, describe the collection location, date and sampling procedures.*
Novel plant genotypes	*Describe the methods by which all novel plant genotypes were produced. This includes those generated by transgenic approaches, gene editing, chemical/radiation-based mutagenesis and hybridization. For transgenic lines, describe the transformation method, the number of independent lines analyzed and the generation upon which experiments were performed. For gene-edited lines, describe the editor used, the endogenous sequence targeted for editing, the targeting guide RNA sequence (if applicable) and how the editor was applied.*
Authentication	*Describe any authentication procedures for each seed stock used or novel genotype generated. Describe any experiments used to assess the effect of a mutation and, where applicable, how potential secondary effects (e.g. second site T-DNA insertions, mosiacism, off-target gene editing) were examined.*

ChIP-seq

Data deposition

☐ Confirm that both raw and final processed data have been deposited in a public database such as GEO.

☐ Confirm that you have deposited or provided access to graph files (e.g. BED files) for the called peaks.

Data access links<br>*May remain private before publication.*	*For "Initial submission" or "Revised version" documents, provide reviewer access links. For your "Final submission" document, provide a link to the deposited data.*
Files in database submission	*Provide a list of all files available in the database submission.*
Genome browser session<br>(e.g. UCSC)	*Provide a link to an anonymized genome browser session for "Initial submission" and "Revised version" documents only, to enable peer review. Write "no longer applicable" for "Final submission" documents.*

Methodology

Replicates	*Describe the experimental replicates, specifying number, type and replicate agreement.*
Sequencing depth	*Describe the sequencing depth for each experiment, providing the total number of reads, uniquely mapped reads, length of reads and whether they were paired- or single-end.*
Antibodies	*Describe the antibodies used for the ChIP-seq experiments; as applicable, provide supplier name, catalog number, clone name, and lot number.*
Peak calling parameters	*Specify the command line program and parameters used for read mapping and peak calling, including the ChIP, control and index files used.*
Data quality	*Describe the methods used to ensure data quality in full detail, including how many peaks are at FDR 5% and above 5-fold enrichment.*
Software	*Describe the software used to collect and analyze the ChIP-seq data. For custom code that has been deposited into a community repository, provide accession details.*

Flow Cytometry

Plots

Confirm that:

☒ The axis labels state the marker and fluorochrome used (e.g. CD4-FITC).

☒ The axis scales are clearly visible. Include numbers along axes only for bottom left plot of group (a 'group' is an analysis of identical markers).

☒ All plots are contour plots with outliers or pseudocolor plots.

☒ A numerical value for number of cells or percentage (with statistics) is provided.

Methodology

Sample preparation	Cells were resuspended in 0.1% BSA in PBS and passed through a 40 μm cell strainer to achieve a uniform single-cell suspension. Cells were stained with DAPI to identify and exclude non-viable cells
Instrument	Cells were solated using a FACSAria II cell sorter (BD Biosciences; RRID:SCR_018934) in the BU Flow Cytometry Core
Software	Flow Jo was used to identify events.
Cell population abundance	Sorted live cells from each treatment group were loaded into individual wells of a 10X Chromium microfluidics chip for single-cell barcoding. Dead cells were excluded.
Gating strategy	DAPI positive cells were defined as dead.

☐ Tick this box to confirm that a figure exemplifying the gating strategy is provided in the Supplementary Information.

Magnetic resonance imaging

Experimental design

Design type	*Indicate task or resting state; event-related or block design.*
Design specifications	*Specify the number of blocks, trials or experimental units per session and/or subject, and specify the length of each trial or block (if trials are blocked) and interval between trials.*
Behavioral performance measures	*State number and/or type of variables recorded (e.g. correct button press, response time) and what statistics were used to establish that the subjects were performing the task as expected (e.g. mean, range, and/or standard deviation across subjects).*

Acquisition

Imaging type(s)	*Specify: functional, structural, diffusion, perfusion.*
Field strength	*Specify in Tesla*
Sequence & imaging parameters	*Specify the pulse sequence type (gradient echo, spin echo, etc.), imaging type (EPI, spiral, etc.), field of view, matrix size, slice thickness, orientation and TE/TR/flip angle.*
Area of acquisition	*State whether a whole brain scan was used OR define the area of acquisition, describing how the region was determined.*

Diffusion MRI ☐ Used ☐ Not used

Preprocessing

Preprocessing software	*Provide detail on software version and revision number and on specific parameters (model/functions, brain extraction, segmentation, smoothing kernel size, etc.).*
Normalization	*If data were normalized/standardized, describe the approach(es): specify linear or non-linear and define image types used for transformation OR indicate that data were not normalized and explain rationale for lack of normalization.*
Normalization template	*Describe the template used for normalization/transformation, specifying subject space or group standardized space (e.g. original Talairach, MNI305, ICBM152) OR indicate that the data were not normalized.*
Noise and artifact removal	*Describe your procedure(s) for artifact and structured noise removal, specifying motion parameters, tissue signals and physiological signals (heart rate, respiration).*

Volume censoring | *Define your software and/or method and criteria for volume censoring, and state the extent of such censoring.*

Statistical modeling & inference

Model type and settings | *Specify type (mass univariate, multivariate, RSA, predictive, etc.) and describe essential details of the model at the first and second levels (e.g. fixed, random or mixed effects; drift or auto-correlation).*

Effect(s) tested | *Define precise effect in terms of the task or stimulus conditions instead of psychological concepts and indicate whether ANOVA or factorial designs were used.*

Specify type of analysis: ☐ Whole brain ☐ ROI-based ☐ Both

Statistic type for inference | *Specify voxel-wise or cluster-wise and report all relevant parameters for cluster-wise methods.*

(See Eklund et al. 2016)

Correction | *Describe the type of correction and how it is obtained for multiple comparisons (e.g. FWE, FDR, permutation or Monte Carlo).*

Models & analysis

n/a	Involved in the study
☐	☐ Functional and/or effective connectivity
☐	☐ Graph analysis
☐	☐ Multivariate modeling or predictive analysis

Functional and/or effective connectivity | *Report the measures of dependence used and the model details (e.g. Pearson correlation, partial correlation, mutual information).*

Graph analysis | *Report the dependent variable and connectivity measure, specifying weighted graph or binarized graph, subject- or group-level, and the global and/or node summaries used (e.g. clustering coefficient, efficiency, etc.).*

Multivariate modeling and predictive analysis | *Specify independent variables, features extraction and dimension reduction, model, training and evaluation metrics.*
